# Effects of In Situ Preheating Technology on Mechanical Properties and Microstructure of FFF-Printed PEEK

**DOI:** 10.3390/mi17030303

**Published:** 2026-02-28

**Authors:** Junhua Wang, Yuanming Mao, Jianan Shen, Yan Lu, Kun Li, Junfei Xu, Zhuangya Zhang, Ruijie Gu, Tancheng Xie

**Affiliations:** 1 School of Mechanical and Electrical Engineering, Henan University of Science and Technology, Luoyang 471003, China; wangjh@haust.edu.cn (J.W.); 18238157404@163.com (Y.M.); 240320010124@stu.haust.edu.cn (J.S.); zhangzhuangya@126.com (Z.Z.); 2 Henan Intelligent Manufacturing Equipment Engineering Technology Research Center, Luoyang 471003, China; 3 Henan Engineering Laboratory of Intelligent Numerical Control Equipment, Luoyang 471003, China; 4 School of Materials Science and Engineering, Henan University of Science and Technology, Luoyang 471023, China; luyan@haust.edu.cn; 5 State Key Laboratory of Mechanical Transmission for Advanced Equipment, College of Mechanical and Vehicle Engineering, Chongqing University, Chongqing 400044, China; kun.li@cqu.edu.cn; 6 College of Mechanical and Vehicle Engineering, Chongqing University, Chongqing 400044, China; mecha_xjf@163.com

**Keywords:** polyether ether ketone, fused filament fabrication, in situ preheating, interlayer bonding, mechanical properties, microstructure

## Abstract

The Polyether ether ketone (PEEK) suffers from insufficient interlayer molecular chain diffusion and weak interfacial fusion during Fused Filament Fabrication (FFF) due to its high melt viscosity and rapid cooling characteristics, restricting the mechanical properties and engineering applications of printed parts. To improve the interlayer bonding quality of FFF-printed PEEK, an in situ preheating technology integrated into the print nozzle was proposed and implemented. Through a high-temperature controllable preheating system that moves synchronously with the nozzle, local precise heating is performed on the surface of the deposited layer to actively regulate the thermal history of the interlayer interface. Systematic studies on the effect of preheating temperature were conducted. The results show that the influence of preheating temperature on part performance follows a trend of first increasing and then decreasing. When the preheating temperature is 280 °C, the comprehensive performance of the specimens is optimal: the tensile strength reaches 69.47 MPa, which is 21.3% higher than that of the non-preheated reference group; the elongation at break is 71.07%; and the porosity decreases to 8.36%. Microstructural analysis reveals that moderate preheating facilitates molecular chain diffusion and interfacial fusion, whereas excessive heating induces thermal oxidative degradation of PEEK, resulting in deteriorated mechanical performance. These findings confirm that in situ preheating represents an effective approach for enhancing interlayer bonding, thereby offering a practical solution for the additive manufacturing of high-performance PEEK components.

## 1. Introduction

Polyether ether ketone (PEEK), as a semi-crystalline high-performance thermoplastic engineering plastic [1], has been widely applied in fields such as aerospace [2], biomedical [3], and automotive industries [4] due to its excellent mechanical properties, high-temperature resistance, chemical stability, and good biocompatibility [5,6,7]. It has become a key material in the field of high-end manufacturing. Fused Filament Fabrication (FFF, a typical Material Extrusion (MEX) additive manufacturing technology), as one of the widely used additive manufacturing technologies [8,9], offers advantages such as low equipment cost, simple operation, and the ability to efficiently fabricate parts with complex geometries [10,11,12]. It demonstrates significant potential for the rapid manufacturing and small-batch production of PEEK components [13].

However, the inherent high melting point, high melt viscosity, and tendency to crystallize of PEEK are in conflict with the layer-by-layer deposition and localized melting characteristics of the FFF process [14,15], leading to the prominent issue of insufficient interlayer bonding strength during fabrication [16]. During FFF printing of PEEK, when the high-temperature PEEK melt extruded from the nozzle is deposited onto the surface of the already solidified, cooler layer, a sharp temperature gradient and rapid cooling solidification occur [17,18]. This results in insufficient diffusion of PEEK molecular chains at the interlayer interface, making it difficult to form effective entanglement and fusion, ultimately leading to weak interfacial bonding [19]. Therefore, weak interlayer bonding has become a key bottleneck restricting the fabrication of high-performance, high-load-bearing PEEK parts using FFF technology [20].

To enhance the interlayer bonding strength of FFF-fabricated PEEK, researchers domestically and internationally have conducted extensive studies primarily focusing on process parameter optimization [21], material modification [22], and post-processing techniques [23]. In terms of printing parameter optimization, research has mostly concentrated on the influence of key process parameters such as nozzle temperature, printing speed, and layer thickness on PEEK printing quality. Liaw et al. [24] found that increasing the nozzle temperature significantly enhances interlayer bonding strength and crystallinity, while shorter waiting times and smaller layer heights help reduce interlayer defects. Gobenas et al. [25] confirmed that nozzle temperature is a critical factor affecting PEEK printing performance, with a suitable range of 380–440 °C; optimization within this range promotes interlayer molecular chain diffusion and crystallinity improvement. Sikder et al. [26] further discovered that a nozzle temperature of 410 °C combined with a chamber temperature of 90 °C can significantly enhance interlayer bonding and reduces pore defects, with microstructural analysis also confirming that high temperatures facilitate molecular chain diffusion. Regarding material modification, researchers have attempted to introduce functional additives or construct blend systems to improve interfacial performance. Li et al. [27] utilized the thermal decomposition of trisilanolphenyl POSS during high-temperature printing to release benzene derivatives, which acted as a plasticizer to promote the diffusion and entanglement of PEEK molecular chains at the interlayer interface, increasing interlayer bonding strength by approximately 20%. Xu et al. [28] investigated the interfacial enhancement mechanism of an aPAEK/PEEK blend system, optimizing interfacial diffusion and strength through component regulation. In post-processing research, techniques such as annealing and hot isostatic pressing have been employed to ameliorate interlayer fusion defects. Wu et al. [29] proposed a powder-assisted hot isostatic pressing post-treatment method using alumina powder as a pressure-transmitting medium. Treating PEEK specimens at 360 °C and 5 MPa resulted in a tensile strength of 102.6 MPa and a crystallinity increase to 42.15%. He et al. [30] systematically studied the effects of different annealing temperatures on the mechanical properties of FDM-printed PEEK, finding that after annealing at 300 °C, tensile, flexural, and compressive strengths increased by 36%, 54%, and 21%, respectively. However, the elongation at break decreased, and internal pores were not completely eliminated.

Although the aforementioned methods have achieved certain success, in-depth analysis reveals that these approaches still have limitations in their mechanisms and ultimate effects. They mostly belong to “passive adaptation” to the printing thermal history or “compensation after fabrication” and do not address the fundamental essence of FFF interlayer bonding [31]—the cross-layer diffusion and entanglement of polymer molecular chains. If the temperature field at the deposition interface cannot be actively controlled, the significant temperature difference between the new melt and the deposited layer will persist [32]. Polymer molecular chains cannot acquire sufficient thermal energy and time to diffuse and entangle across the interface, making it impossible to form strong interlayer bonding after cooling. Consequently, the potential for improving interlayer strength with existing methods is limited.

To further improve the interlayer bonding strength of FFF-printed PEEK, it is imperative to shift from passive process optimization to active regulation of the thermal history at the deposition interface. In response to this need, the present study proposes an active in situ preheating technology. The core of this approach lies in the design and integration of a high-temperature preheating system that moves synchronously with the FFF printhead. Prior to the deposition of each new layer, the system precisely heats the surface of the previously deposited layer, thereby transforming the conventional “hot-cold” interface into a “hot-hot” interface and enabling real-time thermal management during interlayer formation. By investigating the effects of different preheating temperatures on the tensile properties, interlayer fusion state, porosity, and thermal stability of PEEK printed parts, this study reveals the enhancement mechanism of preheating treatment on interlayer bonding, providing new insights and methods for the development of high-performance FFF-printed PEEK technology.

## 2. Design of the In Situ Preheating System

### 2.1. Structural Design of the Preheating System

**Figure 1:**
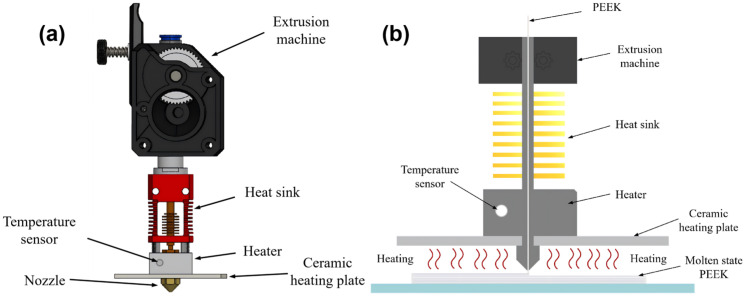
Interlayer in situ preheating system: (**a**) structure diagram of preheating system (**b**) schematic diagram of preheating system.

To achieve active thermal intervention at the interlayer bonding interface during the FFF printing process, this study independently designed an integrated interlayer in situ preheating system incorporated onto the FFF nozzle. The structure of this system is shown in Figure 1. It primarily consists of two core components: a heat source module and a temperature control system. The heat source module utilizes a high-temperature alumina ceramic heating plate with dimensions of 50 mm × 50 mm × 2 mm, a rated power of 100 W, and a maximum operating temperature of up to 500 °C. It is mounted and fixed approximately 3 mm above the printing nozzle, moving synchronously with the printhead to provide following-type preheating for the area about to be deposited. The temperature control system comprises a K-type high-temperature glass fiber thin-film thermocouple (measurement range: −40 °C to 700 °C) and an REX-C100 PID controller. The thermocouple is fixed on the surface of the ceramic heating plate for real-time monitoring of its temperature and is connected to the PID controller to form a closed-loop control system. This setup enables precise regulation of the preheating temperature by adjusting the output power of the ceramic heating plate.

### 2.2. Operating Principle of the Preheating System

The core function of the preheating system lies in actively regulating the thermal environment during the formation of the interlayer interface. Its principle is to proactively provide a controllable additional heat source before depositing each new layer of PEEK melt, performing auxiliary heating on both the newly extruded high-temperature PEEK melt and the underlying cooled deposited layer. By controlling the temperature of this specific area, the initial temperature of the cooled layer is raised while the cooling rate of the PEEK melt is slowed down, thereby reducing the temperature difference between the new and old materials. This elevates the interfacial bonding temperature during the instantaneous fusion stage above the glass transition temperature (Tg, 143 °C) of PEEK and maintains it above this temperature for a longer duration, ensuring sufficient diffusion and entanglement of the polymer molecular chains, thereby improving the interlayer bonding strength of the printed parts [33]. Its specific operating process can be divided into the following four consecutive stages:

(1) Preheating Initiation Stage: Before the printing nozzle moves to the starting position of the predetermined path, the preheating system is activated. The ceramic heating plate begins to heat up to the preset target temperature. Heat is transferred to the surface of the printing layer through both thermal radiation and convection, continuously delivering the generated heat to the surface of the underlying, previously deposited PEEK layer.
(2) Interface Activation Stage: After the deposited layer of the PEEK part absorbs heat from the ceramic heating plate, its temperature gradually increases. When the surface temperature exceeds the glass transition temperature of PEEK, the surface layer of the material transitions from a glassy state to a high-elastic state. This transformation activates the mobility of the molecular chains in the surface layer, changing the material from its original hard state to a soft, viscoelastic state [34,35]. This provides favorable conditions for subsequent interfacial fusion during the deposition of new melt.
(3) Thermal State Preparation Stage: Immediately before the nozzle extrudes the new melt, the preheating system regulates the surface of the deposited layer to an ideal bonding state (i.e., with molecular chains in an activated state). This state alters the contact environment for the new melt, ensuring that the materials to be in contact on both sides of the interface are at a relatively high temperature level. As a result, the original “hot-cold” contact mode is converted into a “hot-hot” contact mode, establishing a thermal condition favorable for molecular chain diffusion across the interface.
(4) Fusion Realization Stage: When the high-temperature new melt is deposited onto the preheated and activated surface of the deposited layer, good thermal contact is formed at the interface. With a reduced temperature gradient and a slower cooling rate, the PEEK molecular chains on both sides of the interface possess high mobility, enabling them to fully diffuse across the interface and entangle with each other. This results in a high-performance printed part.

By enabling precise thermal management throughout the aforementioned stages, the system achieves active intervention in the thermal history of interlayer bonding during the FFF printing process, thereby offering a key technical strategy to fundamentally enhance the interlayer performance of PEEK-printed parts.

## 3. Experimental

### 3.1. Experimental Materials

The Polyether ether ketone (PEEK) filament used in this experiment was provided by Jilin Zhongyan High Polymer Materials Co., Ltd. (Changchun, China), with the grade designation 551G. Its key performance parameters are listed in Table 1. To eliminate the influence of moisture on printing quality, the filament was dried in a blast drying oven (DHG-9070A, Shanghai Yiheng Scientific Instrument Co., Ltd., Shanghai, China) at 120 °C for 5 h before printing. After being cooled to room temperature, it was sealed and stored for later use.

### 3.2. Experimental Equipment

**Figure 2:**
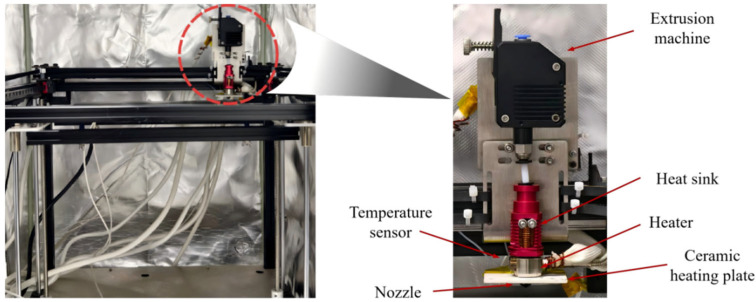
FFF Printing Equipment (The red circle indicates the enlarged view of the nozzle structure).

The printing equipment employed in this experiment was a self-modified high-temperature FFF printer, as shown in Figure 2. Its main frame was constructed from high-strength aluminum alloy. The control system of the printer utilized an MKS Gen-L V2.1 mainboard supporting multiple stepper motors. Both the driving motor and the extrusion motor were 42-type stepper motors. The hot end of the printing nozzle was made of a high-temperature aluminum-copper alloy material. It was equipped with a 70 W power 304 stainless steel heating rod and a PT-1000 platinum resistance temperature sensor (Heraeus, Hanau, Germany) for precise measurement and control of the nozzle temperature. The power supply of the equipment was a switching power supply with a rated voltage of 12 V. Through the coordinated operation of the aforementioned modules, stable extrusion of the PEEK filament was achieved. The in situ preheating system described in Chapter 2 was integrated into this printing platform, constituting a complete experimental fabrication system.

### 3.3. Process Parameters and Specimen Preparation

To achieve univariate controlled experimentation of the preheating temperature, avoid coupling interference from multiple process parameters on the experimental results, and ensure the accuracy and reliability of the conclusions regarding the effect of preheating temperature on the properties of the printed parts, this study fixed the printing speed and other key process parameters based on preliminary experimental results. Considering the certain vertical distance between the nozzle orifice and the lower surface of the ceramic heating plate, a discrepancy exists between the set temperature of the heating plate and the actual temperature at the interlayer interface. Therefore, after calibrating and measuring the interface temperature using a K-type thermocouple and an IR thermal imaging camera (FLIR A615, Wilsonville, OR, USA), four temperature gradients—240 °C, 260 °C, 280 °C, and 300 °C—were ultimately selected as the experimental variables. The specific process parameters are listed in Table 2.

To systematically evaluate the effect of the preheating temperature, a comparative study design was employed, including two comparison dimensions: “with vs. without preheating” and “different preheating temperatures”. The group design is shown in Table 3. Five parallel specimens were prepared for each group to ensure the reliability of the experimental data.

**Figure 3:**
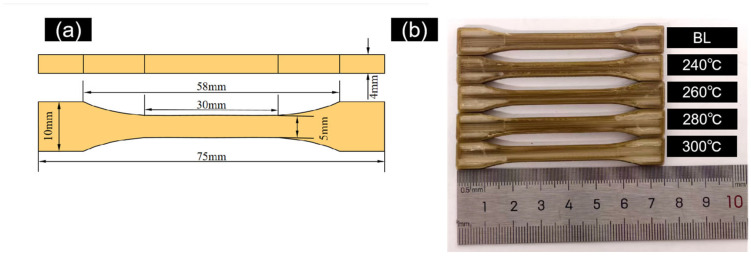
PEEK Tensile Specimens (**a**) Dimension Schematic (**b**) Photograph of FFF-Printed Specimens.

The specimen dimensions conformed to the BA-type tensile specimen specified in GB/T 1040.2-2022 [36], with specific dimensions shown in Figure 3a. First, a 3D model of the specimen was created using SolidWorks 2022 software and exported as an STL file. Subsequently, the model was imported into Ultimaker Cura 5.11 software for layered slicing processing according to the parameters in Table 2, generating G-code files recognizable by the FFF printer. Finally, the FFF system executed the printing task. A photograph of the printed specimens is shown in Figure 3b.

To quantify the thermal effect of the in situ preheating system on the deposited layer and establish a comparable quantitative benchmark for the process, the effective energy density delivered to the unit area of the PEEK substrate layer was calculated based on the Stefan-Boltzmann law of thermal radiation, which is the dominant heat transfer mode of the ceramic heating plate. The calculation formula is as follows:

(1) $$E=\varepsilon \times \sigma \times F\times \alpha \times \left(T_{p}^{4}-T_{0}^{4}\right)\times t$$

where *ε* is the emissivity of the alumina ceramic heating plate, taken as 0.90; *σ* is the Stefan-Boltzmann constant, 5.670373 × 10^−8^ W/(m^2^·K^4^); *F* is the view factor between the heating plate and the substrate layer, taken as 0.85; *α* is the infrared radiation absorption coefficient of PEEK, taken as 0.92; *T_p_* is the absolute temperature of the preheating plate (K); *T*_0_ is the ambient temperature, fixed at 298.15 K (25 °C); *t* is the effective radiation duration on the unit area of the substrate layer, calculated as *t* = 50 mm/35 mm/s ≈ 1.4286 s, where 50 mm is the effective length of the heating plate along the printing direction, and 35 mm/s is the fixed feed rate (printing speed) of this study.

### 3.4. Testing and Characterization Methods

According to ASTM D638 [37] standard, quasi-static tensile tests were performed using an AGX-100kNV2 universal testing machine (Shimadzu, Kyoto, Japan) at room temperature (25 °C), with a gauge length of 25 mm and a tensile speed of 5 mm/min. After the tensile test, sampling for SEM characterization was strictly performed on the main fracture surface of the specimen: a small sample approximately 6 mm in length was cut from the central region of the fracture surface along the direction of the tensile load. The fracture surface perpendicular to the tensile load direction was set as the observation surface to ensure that the observed morphology corresponded to the original fracture surface generated under tensile loading, which directly reflects the tensile failure mechanism of the specimens [38]. The sample surfaces were subjected to gold sputtering treatment (thickness about 5–10 nm), and then observed using a JSM-7800F field emission scanning electron microscope (JEOL Ltd., Tokyo, Japan) to analyze the fracture morphology, failure mode, and interlayer fusion state.

Porosity within FFF-printed parts is a key microscopic defect affecting their mechanical properties, primarily related to insufficient melt flow, entrapped air between layers, and moisture vaporization. In this study, the mass-volume method was used to measure the porosity of the specimens. For measuring porosity, the PEEK specimens were fabricated as rectangular blocks with dimensions of 40 mm × 10 mm × 5 mm. This geometry was chosen to facilitate dimensional measurement. Their preparation method and printing process parameters were identical to those of the tensile specimens. First, a precision electronic balance was used to measure the mass (*M*) of the specimen. Then, a vernier caliper was used to measure its geometric dimensions and calculate its volume (*V*). The theoretical density of solid PEEK was taken as the nominal value provided by the supplier, 1.32 g/cm^3^. The porosity (*P*) was calculated using the following formula:

(2) $$P=\left(1-\frac{M}{V\times \rho_{d}}\right)\times 100\%$$

where *M* is the specimen mass (g), *V* is the specimen volume (cm^3^), and *ρ_d_* is the density of solid PEEK material.

## 4. Results and Discussion

### 4.1. Tensile Properties and Porosity

**Figure 4:**
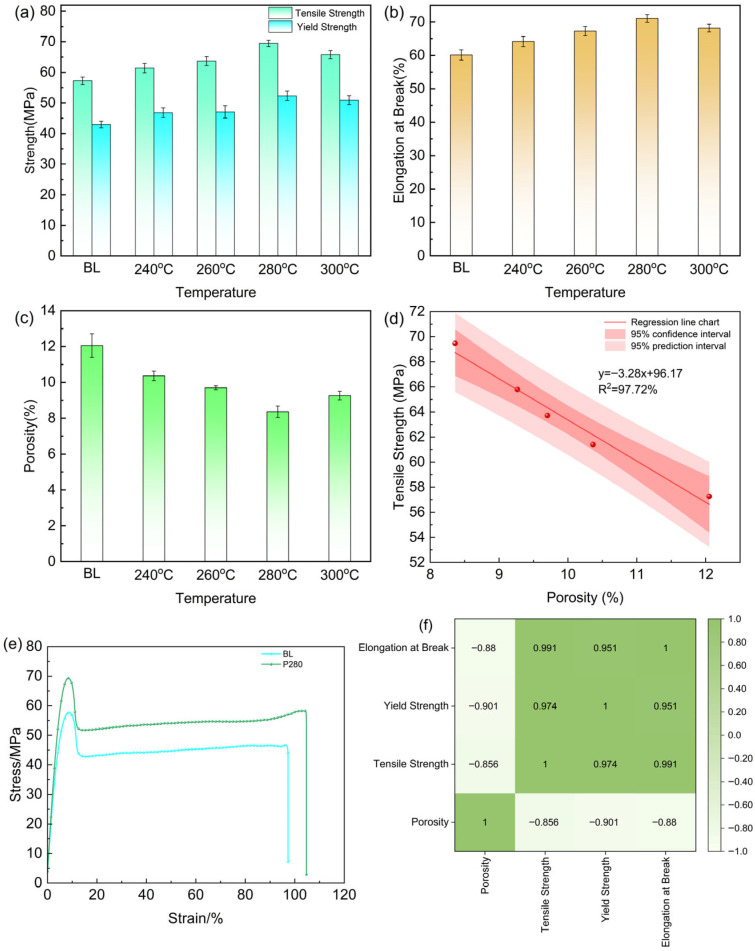
Effect of preheating temperature on the tensile properties of PEEK printed parts: (**a**) Relationship between preheating temperature and tensile strength/yield strength; (**b**) Relationship between preheating temperature and elongation at break; (**c**) Relationship between preheating temperature and porosity; (**d**) Relationship between porosity and tensile strength (The red points represent the measured porosity and tensile strength); (**e**) Stress–strain curves of the BL group and P280 group; (**f**) Correlation coefficient matrix of porosity, tensile strength, yield strength and elongation at break.

The tensile properties and porosity test results of PEEK specimens printed under different preheating temperatures are shown in Table 4, with their variation trends illustrated in Figure 4.

Meanwhile, the effective energy density delivered to the substrate layer under different preheating temperatures was calculated based on the above model, and the results are shown in Table 5.

The results show that the effective energy input increases non-linearly with the rise of preheating temperature, which is consistent with the fourth-power law of thermal radiation. Moreover, a positive correlation was observed between the energy input and the tensile strength of the specimens, underscoring that the increase in preheating energy input serves as the core driver for enhancing interlayer bonding performance.

#### 4.1.1. Variation in Tensile Properties

As shown in Figure 4a, the tensile properties of the PEEK printed parts exhibited a trend of first increasing and then decreasing with the rise in preheating temperature. The baseline group without preheating (BL) showed the lowest tensile strength, only 57.27 MPa. This is attributed to the large temperature gradient at the interlayer interface, leading to insufficient diffusion of PEEK molecular chains, weak interfacial bonding, and the presence of numerous internal pores acting as stress concentration points, causing premature fracture of the specimens.

When the preheating temperature increased to 280 °C, the tensile strength reached a peak value of 69.47 MPa, representing a 21.3% improvement compared to the baseline group. This enhancement is primarily attributed to the effective reduction in the temperature gradient between the new melt and the deposited layer by preheating, which slowed the cooling rate at the interlayer interface. This provided more favorable thermodynamic conditions and a longer time window for cross-interface diffusion and entanglement of the polymer molecular chains. Concurrently, the marked reduction in porosity demonstrates that in situ preheating effectively enhanced interlayer bonding within the printed parts. However, when the preheating temperature was further increased to 300 °C, the tensile strength decreased to 65.79 MPa. This trend indicates the presence of an optimal preheating window, as excessively high temperatures may induce thermal oxidative degradation of the material, partially offsetting the benefits gained from improved interfacial fusion.

The trend of elongation at break with preheating temperature was consistent with that of tensile strength (Figure 4b). The P280 group achieved the maximum value of 71.07%, an 18.2% increase over the baseline group. This result further demonstrates that preheating treatment, by improving the quality of interlayer fusion, enables more uniform plastic deformation of the parts during tensile loading. Stress transfer between layers becomes more continuous and effective, thereby enhancing the overall toughness of the material.

Figure 4e presents the stress–strain curves of the BL and P280 groups. It can be observed that the initial segments of both curves exhibit a linear rise, corresponding to the elastic deformation stage. The stress growth rate of the P280 group is slightly higher than that of the BL group, indicating an improvement in its elastic modulus, which may be attributed to the reduction in interlayer pores and defects resulting from the preheating treatment. The curve of the P280 group lies entirely above that of the BL group, demonstrating that at any given strain value, the P280 group withstands higher stress. This directly evidences that the preheating technology effectively enhances the tensile capacity of the material. The area under the P280 curve (i.e., the fracture energy, representing the material’s ability to absorb energy before fracture) is significantly larger than that of the BL group. This indicates that the P280 specimen consumed more energy prior to fracture, exhibiting markedly superior toughness. In summary, the in situ preheating at 280 °C optimizes the interlayer bonding state, enabling the PEEK printed parts to achieve a significant improvement in strength while maintaining excellent ductility.

#### 4.1.2. Porosity and Its Correlation with Mechanical Properties

Porosity is a key indicator for evaluating internal defects and densification in FFF-printed parts. As shown in Figure 4c, as the preheating temperature increased from no preheating to 280 °C, the specimen porosity decreased from 12.05% to 8.36%. This change indicates that preheating elevates the surface temperature of the deposited layer, bringing it into a high-elastic state. This enhances its adhesion with the new melt, facilitates the expulsion of entrapped air during interfacial fusion, and promotes better bonding between adjacent printed paths, thereby reducing pore defects.

From Figure 4d, a significant negative correlation between porosity and tensile strength is evident (fitted coefficient of determination R^2^ > 0.95). This indicates that reducing internal microscopic defects is one of the core mechanisms by which the preheating process enhances mechanical properties. It is noteworthy that the porosity of the P300 group (9.27%) showed a rebound. This may be related to micropores or microcracks generated by local degradation of PEEK at high temperatures.

As shown in Figure 4f, porosity exhibits negative correlations with all three mechanical properties (r ≈ −0.85 to −0.90). This confirms that internal defects (pores) are a primary factor limiting the mechanical performance of FFF-printed PEEK parts. The reduction in porosity achieved through in situ preheating directly contributes to the enhancement of tensile strength, yield strength, and ductility. Meanwhile, a very strong positive correlation (r > 0.95) was observed among tensile strength, yield strength, and elongation at break. This indicates that under optimal preheating conditions, these properties improve simultaneously rather than trade off—meaning the P280 group not only exhibits higher strength but also enhanced ductility.

### 4.2. Fracture Morphology Analysis

**Figure 5:**
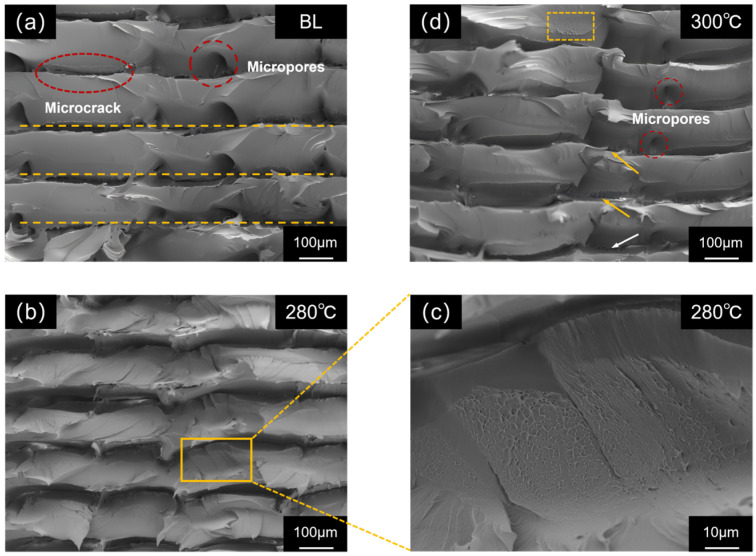
SEM images of tensile fracture surfaces of PEEK printed parts from different groups: (**a**) Baseline group; (**b**,**c**) P280 group; (**d**) P300 group.

To gain deeper insight into the influence of preheating temperature on the fracture mechanism, scanning electron microscopy (SEM) observations were conducted on the tensile fracture surfaces of specimens from each group, with the results shown in Figure 5.

The fracture morphology of the BL group (Figure 5a) exhibits a clear layered structure overall, with a relatively flat fracture surface and small undulations. The boundaries between adjacent deposited filaments (indicated by yellow dashed lines) are distinct and straight, showing obvious micrometer-scale grooves. Numerous pores and microcracks can be observed at interlayer gaps and within the filaments themselves. The formation of these defects can be attributed to two main factors: firstly, air bubbles entrapped during melt deposition were immobilized due to rapid cooling; secondly, unfused regions between layers were torn and amplified during fracture. These microscopic defects readily acted as stress concentration points during tensile loading, leading to specimen failure under relatively low loads. This indicates that under non-preheated conditions, when the newly extruded high-temperature melt is deposited onto the cooled layer surface, the excessive interlayer temperature difference causes extremely rapid cooling, “freezing” the motion of PEEK molecular chains. Effective interdiffusion and entanglement of molecular chains cannot occur between the interfaces of the new and old materials, ultimately resulting in weak interlayer interfacial bonding.

As shown in Figure 5b, the fracture surface of the P280 group (280 °C preheating) was characterized by a rough, continuous, and undulating morphology. Under higher magnification (Figure 5c), a high density of relatively uniform dimple structures with varying depths was visible, features that are hallmarks of microscopic plastic deformation and ductile fracture. The close interconnection of these dimples, along with the significant reduction in micropores and interlayer gaps, points to a high degree of material densification, providing direct evidence for the improved interlayer bonding achieved by preheating. The presence of the dimple structure suggests that stress was transferred more uniformly across the entire specimen cross-section during tensile loading, thereby absorbing more energy, which corresponds to its superior tensile performance. The aforementioned microscopic evidence indicates that 280 °C preheating elevated the surface temperature of the deposited layer above the glass transition temperature of PEEK. This enhanced the mobility of molecular chains on both sides of the interface, providing sufficient time and energy for mutual diffusion and entanglement of molecular chains at the interface between the new and old melts. Preheating effectively reduced the thermal history difference between layers and improved the fusion quality at the interlayer interface.

The fracture surface of the P300 group (300 °C preheating, Figure 5d) displayed complex morphological features, with coexistence of locally smooth and locally rough regions. Although micropores smaller than those in the baseline group were visible between layers (marked by red circles in the figure), and some areas still retained dimple structures similar to the P280 group (marked by yellow squares), indicating that preheating at 300 °C still had a certain positive effect in promoting molecular chain diffusion and plastic deformation, and its fusion degree was superior to the baseline group; However, in other areas, obvious microcracks (marked by yellow arrows) and surface crazing (marked by white arrows) were observed. These defects may have originated from localized chain scission induced by thermal oxidative degradation, thereby creating weak points within the material.

The above analysis reveals a competitive interplay between strengthening and degradation mechanisms during the preheating process: moderate preheating facilitates molecular chain diffusion and improves interlayer interfacial fusion, on the other hand, overheating-induced thermal oxidative degradation introduces microscopic defects.

### 4.3. Mechanism of Preheating and Thermal Stability Analysis

Based on the aforementioned results, the intrinsic mechanism by which preheating temperature affects interlayer bonding strength can be revealed as follows:

(1) Low-Temperature Range (Tp < 280 °C): Within this range, the preheating temperature is insufficient to reach the threshold for active diffusion of molecular chains on the surface of the deposited layer. A significant temperature gradient still exists between the newly deposited melt and the deposited layer, and the cooling rate remains relatively high. Although the mobility of molecular chains is improved compared to the non-preheated condition, the degree of diffusion is still limited, leaving numerous unfused regions at the interlayer interface. This manifests as a moderate improvement in mechanical properties over the baseline group, but porosity remains relatively high, and the extent of performance enhancement is constrained.
(2) Optimal Range (Tp ≈ 280 °C): When the preheating temperature is set to 280 °C, the preheating system maintains the surface of the deposited layer within the temperature range corresponding to the high segmental mobility of PEEK molecular chains. This strategy significantly reduces the temperature gradient across the interlayer interface and slows down the cooling rate of the extruded melt, providing an ideal thermal environment for sufficient interdiffusion and entanglement of molecular chains across the interface. Consequently, a dense and uniform microstructure is formed, which maximizes the enhancement of interlayer bonding strength.
(3) High-Temperature Range (Tp > 280 °C): When the preheating temperature is excessively high (e.g., 300 °C), despite providing even more favorable conditions for thermal diffusion, the PEEK material undergoes thermal oxidative decomposition due to prolonged exposure to a high-temperature oxygen environment. This process leads to molecular chain scission, reducing the number of molecules involved in entanglement at the interface and weakening the interfacial entanglement density. This not only reduces the intrinsic strength of the material but also introduces defects such as microcracks at the interface, leading to a decline in performance and thermal stability.

The thermal history during the FFF printing process directly dictates the crystallization behavior of PEEK. Reducing the interlayer cooling rate through preheating can broaden the cold crystallization window and improve the consistency of crystallization between layers, thereby enhancing the interlayer bonding strength. In this study, although preheating at 240 °C and 260 °C reduced the temperature gradient compared to the non-preheated condition, the thermal energy was still insufficient to elevate the deposited layer surface temperature significantly above the glass transition temperature for an extended duration. Consequently, the molecular chains in the surface layer may not have achieved the mobility required for crystal reorganization, limiting the improvement in crystallinity. In contrast, the optimal preheating temperature of 280 °C maintained the deposited layer surface in a high-elastic state for a prolonged period, promoting more complete molecular chain rearrangement and enhancing crystallinity. This, combined with the observed reduction in porosity and the increase in dimple structures on the fracture surface (indicating better plastic deformation capability, which correlates with a more perfected crystalline structure), suggests that preheating can promote interlayer molecular chain diffusion and crystallization perfection by modulating the thermal history.

Compared with existing thermal management strategies for FFF-printed PEEK, the primary advantage of the nozzle-synchronized in situ preheating proposed in this study lies in its active thermal management of the interlayer interface during the printing process, enabling precise regulation of the interlayer interface temperature. Unlike passive parameter adjustments or post-processing—which either accept the inherent thermal history or attempt remediation after fabrication—this method directly modulates the interfacial thermal history during printing, addressing the core issue of insufficient molecular chain diffusion at the interlayer bond and avoiding the performance trade-offs associated with post-processing routes and the complex formulation design required by material modification approaches. These characteristics endow this technology with significant advantages for manufacturing high-performance PEEK parts.

## 5. Conclusions

To address the issue of weak interlayer bonding in FFF-printed PEEK, this study proposed and implemented a nozzle-integrated in situ preheating technology. The effects of preheating temperature on the mechanical properties and microstructure of PEEK printed parts were systematically investigated. The main conclusions are as follows:

(1) An in situ interlayer preheating system for FFF printing was successfully developed, offering an effective technical solution to the critical issue of weak interlayer bonding in FFF-printed PEEK.
(2) Experimental results demonstrated that preheating temperature influences part performance in a non-monotonic manner, first increasing and then decreasing. An optimal process window was identified at 280 °C, where the specimens exhibited significantly improved interlayer bonding quality and achieved the best comprehensive performance.
(3) The mechanism of the preheating technology was elucidated from a microstructural perspective. Moderate preheating strengthens the interface by slowing the cooling rate and promoting molecular chain diffusion, whereas excessive preheating induces thermal oxidative degradation, leading to performance deterioration.

The in situ preheating technology proposed in this study, which actively regulates the thermal history at the deposition interface during the printing process, offers a new process strategy for additive manufacturing of high-performance polymers and holds significant engineering application value.

## Figures and Tables

**Table 1:** Key Performance Parameters of the PEEK Filament.

Parameter	Specification
Diameter	1.75 mm
Density	1.32 g/cm^3^
Melt Flow Index (MI)	10 g/10 min
Glass Transition Temperature (Tg)	143 °C
Melting Point (Tm)	343 °C
Tensile Strength	100 MPa
Flexural Strength	170 MPa

**Table 2:** FFF Printing Process Parameters.

Parameter Name	Specification
Nozzle Diameter	0.4 mm
Nozzle Temperature	400 °C
Layer Thickness	0.2 mm
Printing Speed	35 mm/s
Line Width	0.4 mm
Infill Density	100%

**Table 3:** Experimental Group Design.

Group Name	Treatment
Baseline Group(BL)	No Preheating
Preheating Experiment Groups	Preheating at 240 °C, 260 °C, 280 °C, 300 °C

**Table 4:** Tensile Properties and Porosity of Specimens from Different Groups.

Group	Preheating Temp. (°C)	Tensile Strength (MPa)	Yield Strength (MPa)	Elongation at Break (%)	Porosity (%)
BL	No Preheating	57.27 ± 1.20	42.94 ± 1.10	60.13 ± 1.54	12.05 ± 0.66
P240	240	61.40 ± 1.53	46.82 ± 1.61	64.13 ± 1.54	10.36 ± 0.26
P260	260	63.72 ± 1.45	47.08 ± 1.99	67.27 ± 1.40	9.71 ± 0.11
P280	280	69.47 ± 1.06	52.35 ± 1.52	71.07 ± 1.18	8.36 ± 0.32
P300	300	65.79 ± 1.36	50.93 ± 1.48	68.17 ± 1.16	9.27 ± 0.24

**Table 5:** Effective Energy Input Delivered to the Substrate Layer of Each Group.

Group	Preheating Temp. (°C)	Effective Energy Density (J/mm^2^)
BL	No preheating	0
P240	240	0.00350
P260	260	0.00416
P280	280	0.00489
P300	300	0.00570

## Data Availability

Data are contained within the article.

## References

[1] Panayotov I.V., Orti V., Cuisinier F., Yachouh J. (2016). Polyetheretherketone (PEEK) for Medical Applications. J. Mater. Sci. Mater. Med..

[2] Danylenko V., Lipovskyi V. (2025). Review of Polymer Fused Deposition Material Additive Manufacturing Technology for Aerospace Application. J. Rocket-Space Technol..

[3] Yang Z., Guo W., Yang W., Song J., Hu W., Wang K. (2025). Polyetheretherketone Biomaterials and Their Current Progress, Modification-Based Biomedical Applications and Future Challenges. Mater. Des..

[4] Dallal S., Eslami B., Tiari S. (2025). Recent Advances in PEEK for Biomedical Applications: A Comprehensive Review of Material Properties, Processing, and Additive Manufacturing. Polymers.

[5] Fang X., Sang L., Zong L., Li Z., Pan Y., Wang C., Zhang H., Wang J., Jian X. (2025). Development of Polyether Ether Ketone-Based Composites by Fused Filament Fabrication: High-Temperature Resistance and High Performance. Compos. Commun..

[6] Chen P., Wang H., Su J., Tian Y., Wen S., Su B., Yang C., Chen B., Zhou K., Yan C. (2022). Recent Advances on High-Performance Polyaryletherketone Materials for Additive Manufacturing. Adv. Mater..

[7] Siraj N., Hashmi S.A.R., Verma S. (2022). State-of-the-art Review on the High-performance Poly (Ether Ether Ketone) Composites for Mechanical, Tribological and Bioactive Characteristics. Polym. Adv. Technol..

[8] Mogan J., Harun W.S.W., Kadirgama K., Ramasamy D., Foudzi F.M., Sulong A.B., Tarlochan F., Ahmad F. (2022). Fused Deposition Modelling of Polymer Composite: A Progress. Polymers.

[9] Xu X., Ren H., Chen S., Luo X., Zhao F., Xiong Y. (2023). Review on Melt Flow Simulations for Thermoplastics and Their Fiber Reinforced Composites in Fused Deposition Modeling. J. Manuf. Process..

[10] Sandanamsamy L., Harun W.S.W., Ishak I., Romlay F.R.M., Kadirgama K., Ramasamy D., Idris S.R.A., Tsumori F. (2023). A Comprehensive Review on Fused Deposition Modelling of Polylactic Acid. Prog. Addit. Manuf..

[11] Rajan K., Samykano M., Kadirgama K., Harun W.S.W., Rahman M.M. (2022). Fused Deposition Modeling: Process, Materials, Parameters, Properties, and Applications. Int. J. Adv. Manuf. Technol..

[12] Borah J., Chandrasekaran M. (2024). Development of ANN Model for Predicting Mechanical Properties of 3D Printed PEEK Polymer Using FDM and Optimization of Process Parameters for Better Mechanical Properties. Phys. Scr..

[13] Ritter T., McNiffe E., Higgins T., Sam-Daliri O., Flanagan T., Walls M., Ghabezi P., Finnegan W., Mitchell S., Harrison N.M. (2023). Design and Modification of a Material Extrusion 3D Printer to Manufacture Functional Gradient PEEK Components. Polymers.

[14] Zanjanijam A.R., Major I., Lyons J.G., Lafont U., Devine D.M. (2020). Fused Filament Fabrication of PEEK: A Review of Process-Structure-Property Relationships. Polymers.

[15] Wang P., Zou B., Xiao H., Ding S., Huang C. (2019). Effects of Printing Parameters of Fused Deposition Modeling on Mechanical Properties, Surface Quality, and Microstructure of PEEK. J. Mater. Process. Technol..

[16] Wang P., Zou B. (2022). Improvement of Heat Treatment Process on Mechanical Properties of FDM 3D-Printed Short- and Continuous-Fiber-Reinforced PEEK Composites. Coatings.

[17] Lee A., Wynn M., Quigley L., Salviato M., Zobeiry N. (2022). Effect of Temperature History during Additive Manufacturing on Crystalline Morphology of PEEK. Adv. Ind. Manuf. Eng..

[18] Liu T., Zhang M., Kang Y., Tian X., Ding J., Li D. (2023). Material Extrusion 3D Printing of Polyether Ether Ketone in Vacuum Environment: Heat Dissipation Mechanism and Performance. Addit. Manuf..

[19] Xu J., He Y., Shi S., Hao S., Tian J., Chen Y., Dai S., Yang C. (2026). Enhancing Interlayer Mechanical Properties of Poly (Ether Ether Ketone) Fused Deposition Modeling Parts by Parameter Optimization and Post-Annealing. Prog. Addit. Manuf..

[20] Wang P., Zou B., Ding S., Li L., Huang C. (2021). Effects of FDM-3D Printing Parameters on Mechanical Properties and Microstructure of CF/PEEK and GF/PEEK. Chin. J. Aeronaut..

[21] El Magri A., El Mabrouk K., Vaudreuil S., Chibane H., Touhami M.E. (2020). Optimization of Printing Parameters for Improvement of Mechanical and Thermal Performances of 3D Printed Poly(Ether Ether Ketone) Parts. J. Appl. Polym. Sci..

[22] Yang Y., Jiang B., Shang Y., Xu Q., He J., Li X., Zhang H. (2023). The Effect of 3D Printing Fluorene-Containing Poly (Aryl Ether Ketone)/Poly (Ether Ketone) Blends on Interlayer Strength. Polymer.

[23] Qu H., Zhang W., Li Z., Hou L., Li G., Fuh J.Y., Wu W. (2022). Influence of Thermal Processing Conditions on Mechanical and Material Properties of 3D Printed Thin-Structures Using PEEK Material. Int. J. Precis. Eng. Manuf..

[24] Liaw C.-Y., Tolbert J.W., Chow L.W., Guvendiren M. (2021). Interlayer Bonding Strength of 3D Printed PEEK Specimens. Soft Matter.

[25] Gobena S.T., Woldeyohannes A.D. (2024). Assessments and Investigation of Process Parameter Impacts on Surface Roughness, Microstructure, Tensile Strength, and Porosity of 3D Printed Polyetherether Ketone (PEEK) Materials. Results Eng..

[26] Sikder P., Challa B.T., Gummadi S.K. (2022). A Comprehensive Analysis on the Processing-Structure-Property Relationships of FDM-Based 3-D Printed Polyetheretherketone (PEEK) Structures. Materialia.

[27] Li Q., Zhao W., Niu B., Wang Y., Wu X., Ji J., Li Y., Zhao T., Li H., Wang G. (2021). 3D Printing High Interfacial Bonding Polyether Ether Ketone Components via Pyrolysis Reactions. Mater. Des..

[28] Xu Q., Xu W., Yang Y., Yin X., Zhou C., Han J., Li X., Shang Y., Zhang H. (2022). Enhanced Interlayer Strength in 3D Printed Poly (Ether Ether Ketone) Parts. Addit. Manuf..

[29] Wu W., Xin J., Hu B., Chen R., Huang D., Huang Z., Feng J., Du C., Shan B. (2023). Achieving Injection Molding Interlayer Strength via Powder Assisted Hot Isostatic Pressing in Material Extrusion Polyetheretherketone. Addit. Manuf..

[30] He Y., Shen M., Wang Q., Wang T., Pei X. (2023). Effects of FDM Parameters and Annealing on the Mechanical and Tribological Properties of PEEK. Compos. Struct..

[31] Liparoti S., Sofia D., Romano A., Marra F., Pantani R. (2021). Fused Filament Deposition of PLA: The Role of Interlayer Adhesion in the Mechanical Performances. Polymers.

[32] Wolszczak P., Lygas K., Paszko M., Wach R.A. (2018). Heat Distribution in Material during Fused Deposition Modelling. Ring Politischer Jugend.

[33] Omer M.A.E., Shaban I.A., Mourad A.-H., Hegab H. (2025). Advances in Interlayer Bonding in Fused Deposition Modelling: A Comprehensive Review. Virtual Phys. Prototyp..

[34] Matveenko V.P., Smetannikov O.Y., Trufanov N.A., Shardakov I.N. (2012). Models of Thermomechanical Behavior of Polymeric Materials Undergoing Glass Transition. Acta Mech..

[35] Liu G., Hu N., Huang J., Tu Q., Xu F. (2024). Experimental Investigation on the Mechanical and Dynamic Thermomechanical Properties of Polyether Ether Ketone Based on Fused Deposition Modeling. Polymers.

[36] (2022). Plastics-Determination of Tensile Properties-Part 2: Test Conditions for Moulding and Extrusion Plastics.

[37] (2022). Standard Test Method for Tensile Properties of Plastics.

[38] Li T., Yang Z., Cui J., Chen W., Almatani R., Wu Y. (2025). Prediction and Optimization of Stretch Flangeability of Advanced High Strength Steels Utilizing Machine Learning Approaches. Sci. Rep..

